# Stiffness memory of EA.hy926 endothelial cells in response to chronic hyperglycemia

**DOI:** 10.1186/1475-2840-12-96

**Published:** 2013-06-27

**Authors:** Marta Targosz-Korecka, Grzegorz D Brzezinka, Katarzyna E Malek, Ewa Stȩpień, Marek Szymonski

**Affiliations:** 1 Research Centre for Nanometer-Scale Science and Advanced Materials, NANOSAM, Faculty of Physics, Astronomy, and Applied Computer Science, Jagiellonian University, Reymonta 4, 30-059 Krakow, Poland; 2 Genetic Diagnostics and Nutrigenomic Unit, Department of Clinical Biochemistry, Jagiellonian University Medical College, Krakow, Poland

**Keywords:** Endothelial cells, Glycemic memory, Cell stiffness, Atomic force microscope, Hyperglycemia, Cardiovascular complications

## Abstract

**Background:**

Glycemic memory of endothelial cells is an effect of long-lasting hyperglycemia and is a cause of various diabetics complications, that arises despite of the treatment targeted towards returning low glucose level in blood system. On the other hand, endothelial dysfunction, which is believed to be a main cause of cardiovascular complications, is exhibited in the changes of mechanical properties of cells. Although formation of the glycemic memory was widely investigated, its impact on the mechanical properties of endothelial cells has not been studied yet.

**Methods:**

In this study, nanoindentaion with a tip of an atomic force microscope was used to probe the long-term changes (through 26 passages, c.a. 80 days) in mechanical properties of EA.hy926 endothelial cells cultured in hyperglycemic conditions. As a complementary method, alterations in the structure of actin cytoskeleton were visualized by fluorescent staining of F-actin.

**Results:**

We observed a gradual stiffening of the cells up to 20th passage for cells cultured in high glucose (25 mM). Fluorescence imaging has revealed that this behavior resulted from systematic remodeling of the actin cytoskeleton. In further passages, a drop in stiffness had occurred. The most interesting finding was recorded for cells transferred after 14 passages from high glucose to normal glucose conditions (5mM). After the transfer, the initial drop in stiffness was followed by a return of the cell stiffness to the value previously observed for cells cultured constantly in high glucose

**Conclusions:**

Our results indicate that glycemic memory causes irreversible changes in stiffness of endothelial cells. The formation of the observed “stiffness memory” could be important in the context of vascular complications which develop despite the normalization of the glucose level.

## Background

Hyperglycemia is one of the main factors, which induce dysfunction of endothelial cells (ECs). It can lead to an increase in the permeability of the endothelial monolayer and to the development of vascular diseases such as hypertension, arteriosclerosis or retinopathy [1-3]. In ECs, contrary to most of other cells, the increased extracellular glucose level is not accompanied by a decrease in the rate of glucose transmembrane transport. This mechanism makes ECs especially sensitive to elevated glucose level and makes them a major target of hyperglycemia induced dysfunction [4]. Increased intracellular glucose concentration triggers processes specific for oxidative stress and/or pro-inflammatory state, in particular an increase in the secretion of mitochondrial reactive oxygen species (ROS) [5-8]. As described by Yao and Brownlee [6], overproduction of ROS is one of the key steps in the pathogenic process leading to cardiovascular complications in diabetic patients. Consecutive and long-lasting influence of high glucose (chronic hyperglycemia) on ECs evokes dysfunctional paracrine mechanisms and induces epigenetic changes that can accelerate cell aging [9-11]. It seems that such changes cannot be reversed by a simple normalization of glucose level. This effect is referred to as glycemic (or metabolic) memory and is responsible for various complications in the treatment of diabetic patients. First findings of glycemic memory were reported over 20 years ago and later on confirmed basing on large clinical trials [2,12,13].

It was postulated that alterations in mechanical properties of ECs are directly responsible for controlling the permeability of endothelium and for processes that occur during shear stress [14]. In particular, a so called stiff endothelium cell syndrome (SECS) can lead to arterial hypertension and atherosclerosis [15]. In addition, in recent years, there is a growing number of papers validating cell stiffness as an indicator of ECs functional state [16-19]. For example, it has been proven that the eNOS release is modulated by ECs stiffness, i.e., NO release follows endothelial nanomechanics, but not vice versa [18]. Our previous studies on the inflammatory influence of TNF- *α* on ECs [19] showed that monitoring the morphology and nanomechanics of the cells provides a deep insight into the primary stage of endothelial dysfunction. Although glycemic memory is extensively studied at the epigenetic and cellular levels (histones modifications and mitochondria metabolism), the long-term effect of hyperglycemia on cell stiffness has never been investigated on living cells. To our knowledge, until now experiments studying hyperglycemia effects on ECs stiffness have been performed only for fixed cells transferred for measurements to the air conditions [20] and thus such measurement have a limited bioclinical relevance.

In the presented report we focus on the influence of chronic hyperglycemic conditions on the mechanical properties of ECs. *In vitro* nanoindentation spectroscopy with an AFM tip was used to characterize the changes in elastic properties of permanent ECs line (EA.hy926) cultured in high glucose (25 mM - HG) and transferred to normal (5 mM - NG) glucose concentrations. The main purpose of this study was to investigate whether glycemic memory will be reflected in mechanical properties of the cells transferred to NG culture after long-time exposure to HG. In order to observe long-term changes in the cell elasticity, experiments were carried out on the permanent ECs line EA.hy926, which is believed to represent an adequate cellular model in various diabetic complications [21]. Moreover, since the dedicated medium for culturing these cells contains high glucose concentration [22], this cell line is well suited for studying the chronic hyperglycemia effects.

## Methods

### Culture of EA.hy 926

EA.hy 926 is a permanent cell line derived by fusing human umbilical vein endothelial cells with cell line A549 [23]. The culture was maintained according to the protocol provided by the distributor of the cell line (ATCC cat. CRL 2922, Manassas, Virginia, USA). EA.hy926 cells were cultured in DMEM medium with 25 mM glucose (HG) supplemented with 10% fetal bovine serum (cat. No 10082-147, Invitrogen), 2 mM L-glutamine, and 2% HAT supplement (cat. 21060-017, Invitrogen) [22]. After 14 passages (P14 HG), the whole set of cells was divided into two groups cultured in parallel: the first one was still kept in the conditions described above; the second one was cultured in DMEM with 5 mM glucose (NG), supplemented as above.

### Sample preparation for stiffness measurements

The glass slides were cleaved into 1 cm x 1 cm plates, rinsed both sides with ethanol and exposed to UV light in laminar chamber for 30 min in order to sterilize. Next, EA.hy926 cells were seeded on the glass slides at density of 10^4^ cells/ml, and cultured to 80% confluence. Afterwards, the sample was immediately taken for measurements.

### Nanoindentation measurements using an AFM tip

AFM measurements were performed with a commercial instrument Nanoscope IIIa Multimode SPM (Veeco Instruments, Santa Barbara, CA, USA). All experiments were performed on unfixed ECs in DMEM solution using a commercial fluid chamber (Veeco Instruments). V-shape gold-coated cantilevers (MLCT multilever, Veeco Probes, Camarillo, CA, USA) with nominal spring constants of 0.01 N/m were used for nanoindentation measurements. The spring constant of each cantilever was calculated basing on the thermal oscillations spectrum analyzed using NanoScope 6.1 software, while the tip radius was estimated basing on a scan of a test gird (TGT-1, NT-MDT).

**Figure 1:**
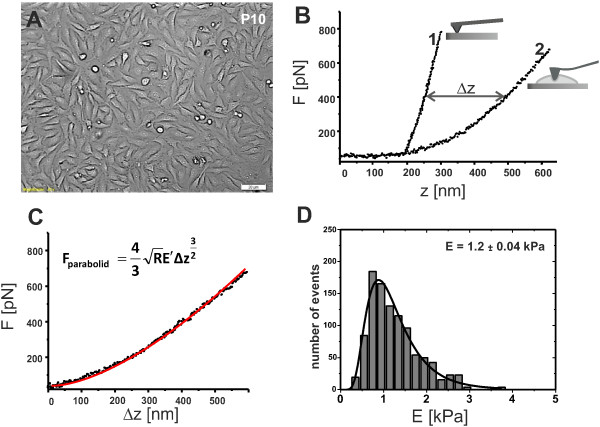
**Methodology of the measurement of elasticity parameter by means of nanoindentation with an AFM tip A)** An optical preview of the sample helps to monitor the experimental conditions and to choose the measurement area over the cell.**B)** Sample force-distance curves, 1 - reference curve acquired at a control surface (glass), 2 - force-distance curve taken at the cell. **C)** The indentation curve obtained on the basis of 1 and 2. The value of the elasticity parameter is evaluated for each curve, according to the model proposed by Sneddon for a paraboloidal tip [24]. **D)** Histogram presenting distribution of the elasticity parameter obtained from the analysis of approx. 1200 indentation curves acquired at a single sample, from multiple cells. The mean value is calculated from the log-normal distribution fit.

Figure 1A shows the optical image of the monolayer formed by cells during the experiment. For a given passage, two samples were examined, about 12-14 cells for each sample, chosen randomly. The time of characterization of each sample was limited to 1.5 h. In the nanoindentation spectroscopy, the AFM tip is attached to a flexible cantilever and acts as an indenter. Using a piezo-scanner, a cell placed on a stiff substrate is pushed towards the tip. This leads to a gentle indentation (deformation) of the cell. The resulting elastic force (*F*) applied to the cantilever (related to its bending) is measured as a function of the scanner position (*z*) - this dependence is visualized on a so called force-distance curve. Later, force-indentation curve [*F*(*δz*)] is obtained from two separately measured force-distance curves [*F*(*z*)]. The first force-distance curve, measured on the cell, contains the information about both the elastic properties of the cell and the cantilever (see Figure 1B). Since the AFM hardware only controls the relative scanner position, the indentation depth *δz* cannot be directly determined from a single measurement. Therefore, for a reference, a second force-distance curve is measured on a significantly stiffer substrate (glass), for which the elastic deformation is negligible and the cantilever bending is solely determined by the position of the piezo-scanner (Figure 1B). Comparison of curves acquired for the cell and for the stiff substrate allows to obtain the indentation depth *δz* and plot the final force-indentation curve (see Figure 1C). Figure 1D presents histograms of the elasticity parameter value calculated basing on force-indentation curves acquired for 8 cells. In order to evaluate the most probable value of the elasticity parameter, a Log-normal function was fitted to the histogram values.

### Actin cytoskeleton imaging

For fluorescence microscopy, cells were seeded on 24-well plates in the same concentration as described above. Before staining, the cells were rinsed with pre-warmed PBS and then fixed with 2.5% formaldehyde for 10 minutes. After rinsing them again, cells were permeabilized with 0.1% Triton X for 4 minutes. Next, cells were incubated in PBS with 1% bovine serum albumin (BSA) for 30 minutes and immediately exposed to phalloidyne conjugated with Alexa Fluor (Invitrogen) for 20 minutes. Prior to the measurement, wells were rinsed twice with PBS. Images were obtained by Olympus IX71 through 40x objective recorded and processed with Olympus Cell Sense software. Fluorescence was excited with Olympus X-Cite Q120 lamp and detected by Olympus U-MWIB2 (Phallodoxine) emission filters.

## Results

### AFM measurements of the cell elasticity parameter

Main motivation of our experiment was to investigate the elastic properties of EA.hy926 ECs cultured in HG conditions and after the transfer of the cells from HG to NG. The base culturing medium for this cell line is the high glucose medium, hence the first part of the experiment was carried out for cells cultured in HG. Measurements were continued until the cells incubated in HG conditions lost the ability to proliferate, which occurred at passage P26-P27. At the time of half achievable passages - after P14, we intentionally changed the concentration of HG to NG for half of the cells, while continuing with HG for the other half.

**Figure 2:**
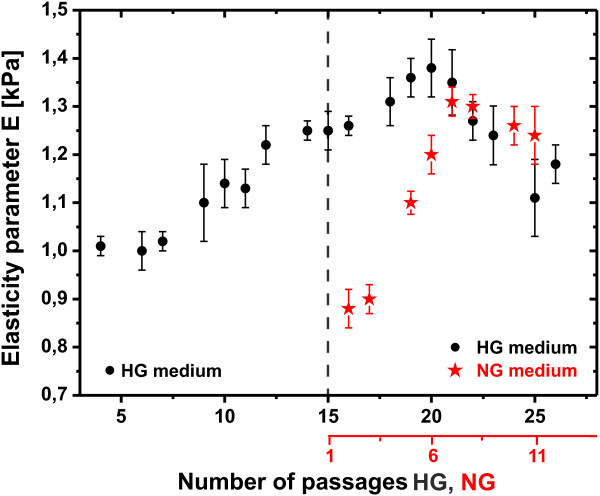
**Changes of elasticity parameter for subsequent passages of the endothelial cells (c.a. 80 days).** Results for cells cultured in HG (25 mM glucose concentration) are marked with black dots. Red asterisks depict values obtained for cells moved from HG to NG (5 mM glucose concentration) conditions. Each point on the graph represents the mean elasticity parameter and error bars correspond to its standard deviation obtained from the log-normal distribution fit to approx. 1200 indentation curves.

The changes of elastic properties for consecutive passages of EC are presented in Figure 2. Each point on the graph corresponds to the mean elasticity parameter value obtained from the analysis of c.a. 1200 indentation curves (12-14 cells) collected for each passage. Black dots represent results obtained for cells cultured in HG medium (glucose concentration 25 mM). There is a distinct stiffening trend up to P20. It can be observed, that the elasticity parameter does not increase smoothly, but rather in a stepwise manner - after each relevant growth in stiffness (i.e. P4-P7, P10-P13, P14-P16), the value remains similar in the few subsequent passages. In late passages (higher than P21), the elasticity parameter decreases with time.

After P14, a subpopulation of the cells was transferred to medium with normal glucose level (NG) (5 mM). The results of elasticity measurements for those cells are marked in Figure 2 with red asterisks. Before the transfer to NG conditions, the elasticity parameter value was (1.24±0.02) kPa. Initially, the exchange of culturing conditions results in a distinct drop, to (0.88±0.04) kPa, in elasticity parameter, *i.e.*, cells become softer. However, maintaining the culture in NG for 4 additional passages results in the return of the elastic properties to the value observed before the change of glucose concentration and very close to the value characteristic for cells cultured constantly in HG.

The latter observation indicates that culturing the cells in hyperglycemic environment develops persistent modification of elastic properties, which cannot be reverted by a simple decrease in glucose concentration, even to the physiological level.

### Fluorescence imaging

**Figure 3:**
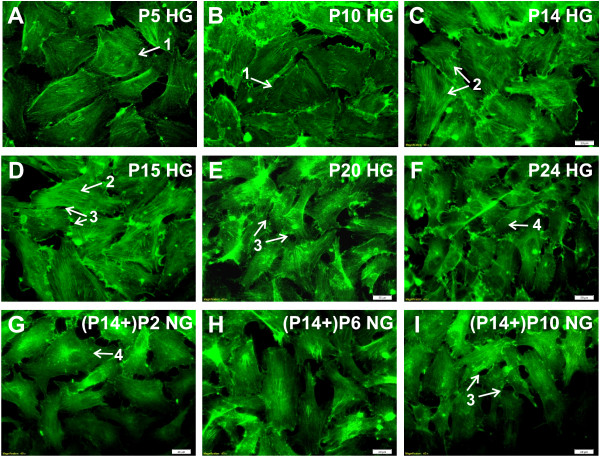
**Fluorescent staining of F-actin.** Cells cultured in HG medium: P5 **(A)**, P10 **(B)**, P14 **(C)**, P15 **(D)**, P20 **(E)**, P24 **(F)** and for the cells moved to NG after 14 passages in hyperglycemic environment (P14+)P2 NG **(G)**, (P14+)P6 NG **(H)**, (P14+)P10 NG **(I)**. The images are organized in such a way, that images **(D)**, **(E)**, **(F)** can be easily compared to **(G)**, **(H)**, **(I)**, respectively. Initially, cell present normal structure of actin cytoskeleton (arrow 1). Later, the polymerization of F-actin leads to stress fibers formation (arrow 2). Additionally, numerous intercellular gaps are formed (arrow 3). Arrow 4 indicate visible actin depolymerization. Images are representative of n=20 independent experiments.

Elasticity measurements were supplemented with fluorescent cytoskeleton staining of actin fibers (F-actin). Representative images for selected HG passages are presented in Figure 3: P5 (A), P10 (B), P14 (C). A normal (unchanged) cytoskeleton structure could be observed in cells cultured up to 10 subsequent passages in hyperglycemic conditions (Figure 3A). After 14 passages, an increase in the F-actin content is observed. This indicates that hyperglycemia stimulates both polymerization of F-actin and stress fibers formation. Moreover, it is visible, that after P14 HG the modification in the structure of the cortical actin cytoskeleton leads to the intercellular gap formation and thus to the increase in the permeability of the endothelium.

After P20 the ECs layer becomes less cohesive and the integrity of endothelial barrier decreases significantly: cells are shrunk and numerous gaps between them are present. As depicted in Figure 2D and 2E, the shape of cells changes: they become elongated, F-actin fibers are formed along the cell, across its central part. These observations are typical for dysfunctional endothelium - ROS production and activation of intracellular pathways contributes to the intercellular gaps formation [25]. Lowering the glucose concentration to 5 mM after initial 14 passages in HG, caused significant changes in the organization of cytoskeleton, as presented in Figures 3G-I. Initial depolymerization of F-actin in (P14+)P2 (Figure 3G) is reverted in (P14+)P6 (Figure 3F) to the typical HG pattern observed in the passage P20 HG-P24 HG. Such changes correlate with the changes of elasticity parameter presented in Figure 2.

## Discussion

We investigated the mechanical properties of ECs during 26 subsequent passages (ca. 80 days) in two scenarios: ECs cultured constantly in hyperglycemic conditions and ECs reverted to normal glucose level after initial exposition to high glucose. Our results revealed that long-term influence of hyperglycemia leads to distinct alterations in the elastic properties of ECs and in subsequent modifications of the cellular monolayer. First of all, for ECs incubated in HG, we observe a stepwise increase in the cell stiffness up to P20. Literature data unambiguously indicate that overproduction of mitochondrial ROS is one of the main factors of hyperglycemia-induced endothelium damage [5-7,13,26]. On the other hand, it has been well established that ROS production decreases the bioavailability of NO produced by eNOS [13]. Basing on the correlation between NO production and cell stiffness described in [18,19], we can postulate that elevated ROS production in hyperglycemic condition is accompanied by an increase in ECs stiffness. Moreover, the gradual stiffening of cells is a reflection of progressive endothelium dysfunction. The observation discussed above is well supported by our fluorescence images, that present the structure of actin cytoskeleton, which is the main parameter determining the stiffness of cells [15]. For example, the initial increase in cell stiffness observed with AFM up to P14 is related to the polymerization of actin filaments and the formation of stress fibers (Figures 3A, B, C). Moreover, it has been shown that the F-actin to G-actin ratio is elevated in cells with an increased ROS production [27]. The latter fact supports our conclusion: the changes in ECs stiffness and F-actin polymerization are related to a gradual increase in ROS production by cells cultured in hyperglycemic conditions.

Consequently, for P20 (ECs cultured in HG), a maximal value of cell stiffness can be observed. After this passage, a gradual decrease in ECs stiffness took place. Most likely, this distinct change originates from oxidative stress, which results in cell damage or in accelerated aging [11]. It was reported, that aged endothelium is characterized by an increased cell membrane motility and production of a large number of protrusions or microparticles [28]. The progressing depolymerization of F-actin, visible in fluorescence images, may be interpreted as a destabilization of cytoskeleton. We propose, therefore, that the decrease in stiffness is caused by the oxidative stress.

Now, let us discuss the alterations in elastic properties of ECs transferred from HG to NG. Initially, after the switch to NG medium, the cells rapidly became more elastic, from (1.24±0.02) kPa to (0.88±0.04) kPa. This quick response could be attributed to the metabolic shock triggered by lowering glucose concentration. Moreover, the fluorescence images (Figure 3D and G) show that depolymerization process is consistent with our cell stiffness measurements.

What is even more important, after next 6 passages in NG medium, the return to the level of stiffness specific for cells permanently cultured in HG is observed. In other words, after P20 HG and (P14+)P6 NG, cells show very similar elastic properties. This means that lowering glucose to the normal level (NG) does not bring the reversal of the mechanical properties. Hence, we introduce the term “stiffness memory” as a phenomenon of permanent change in the mechanical properties of ECs in chronic hyperglycemia.

Most probably the detected irreversible change in the elastic properties of ECs is a direct manifestation of glycemic memory. Hence, stiffness memory, similarly to glycemic memory, may be related to elevated mitochondrial ROS production, which is not inhibited after glucose normalization. As postulated in [29], chronic hyperglycemia can modify the function of mitochondria via glycation of mitochondrial proteins and may induce prolonged overproduction of mitochondrial ROS.

The phenomenon of stiffness memory enriches the understanding of vascular complications, which originate from the glycemic memory. Recently, in a wide review Jermendy [30] stated that basing on the numerous clinical evidence glycemic memory is considered by clinicians as an existing phenomenon, not just a hypothesis. The observed stiffness memory may be a part of the vascular memory observed in different clinical trials reviewed in [30].

This work was carried out for a permanent EA.hy926 cell line. Further work on primary EC lines is required to get a more detailed insight into the molecular mechanism of the stiffness memory effect. It would be also very interesting to check whether this kind of memory can be “switched-off” in a similar way as the glycemic memory effect i.e. by the simultaneous application of mitochondrial antioxidants (decrease of ROS production) and reduction of glucose concentration [31].

## Conclusions

This report discussed the role of chronic hyperglycemia in the modification of ECs mechanical properties.It has been reported previously that increased production of ROS is the key factor linking hyperglycemia and diabetes complications. We believe that formation of the stiffness memory, which alters the global mechanical properties of the endothelial monolayer, reveals a novel aspect of the phenomenon of the glycemic memory. Our findings underline the impact of glycemic memory on the mechanical properties of endothelium with its significant implications for the development of vascular complications caused by chronic hyperglycemia.

## Abbreviations

EC: Endothelial Cell; NO: Nitric Oxide; TNF-α: Tumor Necrosis Factor Alpha; : ; HG: High Glucose; DMEM: Dulbecco‘s Modified Eagle Medium; PBS: Phosphate Buffered Saline; AFM: Atomic Force Microscope.

## Competing interests

The authors declare that they have no competing interests.

## Authors’ contributions

MTK researched the data, wrote the manuscript and contributed to the discussion, GDB wrote the manuscript and contributed to discussion, KEM researched the data, ES reviewed the manuscript and contributed to discussion, MS reviewed the manuscript and contributed to discussion. MTK is the guarantor of this work and, as such, had full access to all the data in the study and takes responsibility for the integrity of the data and the accuracy of the data analysis. All authors read and approved the final manuscript.
